# Genetic Basis of Egg Production in Baicheng-You Chickens: A Genome-Wide Association Study

**DOI:** 10.3390/ani15233360

**Published:** 2025-11-21

**Authors:** Gaoyun You, Tinghao Jiang, Haiying Li, Wei Dong, Xiaoyu Zhao, Shihao Zhang, Zhuocheng Hou, Herong Liao, Zhongtao Yin

**Affiliations:** 1 College of Animal Science, Xinjiang Agricultural University, Urumqi 830091, China; 17628603149@163.com (G.Y.); jth_007@163.com (T.J.); vz_zxy@163.com (X.Z.); 2 Xinjiang Nuqibai City You Chicken Development Co., Ltd., Aksu 842300, China; 18095870823@163.com (W.D.); 13579773681@163.com (H.L.); 3 College of Animal Science and Technology, China Agricultural University, Haidian, Beijing 100193, China; sy20243040975@cau.edu.cn (S.Z.); zchou@cau.edu.cn (Z.H.)

**Keywords:** Baicheng-You chicken, egg production performance, whole genome sequencing, genome-wide association study

## Abstract

The Baicheng-You chicken is a valuable local breed in Xinjiang, China, known for its strong disease and stress resistance and excellent meat quality. However, it has not been scientifically bred or protected, leading to low production performance, particularly in reproductive traits. This study collected phenotypic and whole-genome resequencing data from 742 individuals at an average coverage of −5×, constructing a comprehensive variation map for the first time. Heritability estimates for egg-laying traits were moderate to high, ranging from 0.382 to 0.592. Through genome-wide association analysis, 19 SNPs and 120 candidate genes potentially associated with egg-laying traits were identified, with NCAPG, FAM184B, and the HOXA family playing crucial roles in regulating reproductive performance. These findings enable marker-assisted selection for higher egg yield, offering a targeted strategy to enhance productivity while preserving the genetic diversity and adaptive traits of this local breed.

## Simple Summary

The Baicheng-You chicken is a valuable local breed in Xinjiang, China, known for its strong disease resistance and excellent meat quality. However, it has not been scientifically bred, leading to low egg production, which limits its economic value and conservation. This study aimed to understand the genetic basis of egg-laying traits to support breeding improvements. We collected egg-laying records and blood samples from 742 hens, using whole-genome sequencing to identify genetic variations. We found that traits like the number of eggs laid and the maximum consecutive laying days are moderately to highly heritable, meaning they can be improved through selective breeding. We identified 19 genetic markers and 120 candidate genes associated with egg production, including genes known to influence growth and reproduction in other animals. These results provide the first genetic map for this breed, offering practical markers for breeders to select chickens with better egg-laying performance. This work helps preserve the Baicheng-You chicken as a genetic resource while enhancing its productivity for sustainable farming.

## 1. Introduction

Chickens are among the most successfully domesticated animals, providing high-quality protein through their meat and eggs to consumers worldwide. The Baicheng-You chicken, a valuable local breed from Xinjiang, China, is renowned for its strong disease resistance, tender meat, and significant subcutaneous fat deposition [1]. Balancing growth with improved meat quality and reproductive performance has been a persistent challenge in poultry breeding. Unlike commercial breeds, the Baicheng-You chicken has not undergone intensive artificial selection. Despite its superior flavor and quality, its poor growth performance hinders sustainable development. Notably, although productive under improved management, with an annual yield of 160–180 eggs, the Baicheng-You chicken’s egg production remains substantially lower than the 300+ eggs typically achieved by modern commercial laying breeds [2]. Currently, the lack of genetic parameter estimation and molecular marker discovery for egg-laying traits in the Baicheng-You chicken impedes the conservation of genetic resources and enhancement of production performance.

Decades of research in genetic theory and breeding techniques have led to effective selection and substantial improvements in egg-laying traits, culminating in the development of distinctive commercial egg-laying breeds [3,4]. Previous studies have revealed that egg production in chickens is precisely regulated by genes [5,6]. The whole-genome resequencing data have shown that core traits such as egg number and production rate in breeds like Hy-Line and Shuanglian exhibit medium-to-high heritability [7,8], while the age at the start of laying shows medium-to-low heritability [9,10]. Functional genomics and population genetics approaches have identified key genes and variants, such as BMP15 [11,12] and GDF9 [13], that regulate egg production traits, significantly advancing breeding progress. Therefore, estimating genetic parameters for egg production-related traits (e.g., heritability and genetic correlations) using whole-genome resequencing data will guide optimal breeding strategies by identifying the most promising traits for genetic improvement. Concurrently, identifying functional genes and genetic variants through genome-wide association analysis will provide molecular tools for marker-assisted selection, enabling more efficient genetic enhancement while preserving the breed’s unique genetic diversity.

We hypothesized that the genetic architecture of egg-laying traits in the Baicheng-You chicken could be elucidated through a genome-wide approach, thereby uncovering significant genetic markers and potential candidate genes.

To test this hypothesis and address the existing knowledge gap, we established an experimental population of 742 Baicheng-You Chickens and employed high-depth whole-genome resequencing with the following objectives: to construct a comprehensive genome-wide variation map, to estimate the genetic parameters of five key egg-laying traits, and to identify associated SNPs and candidate genes through a genome-wide association study. This work aims to provide essential genetic resources for the scientific conservation and molecular breeding of this unique breed.

## 2. Materials and Methods

### 2.1. Ethical Statement

All experimental procedures involving animals were approved by the Animal Welfare and Ethics Committee (2023007) of Xinjiang Agricultural University, Urumqi, Xinjiang, China.

### 2.2. Experimental Animals and Phenotypic Measurements

**Figure 1:**
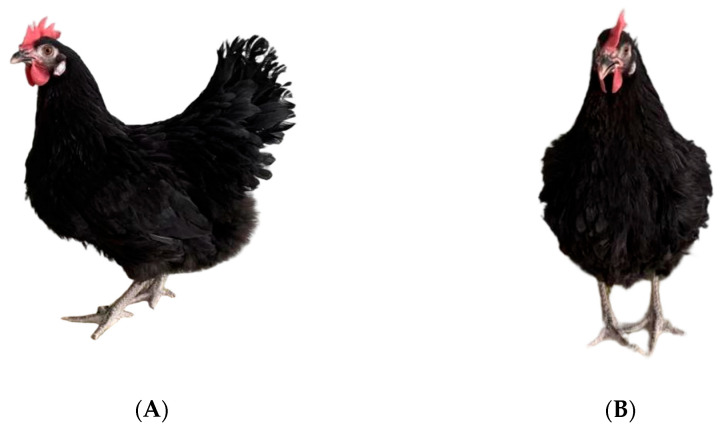
Representative photographic images of Baicheng-You chickens. (**A**) Side and (**B**) front views of an adult hen.

This experiment was performed on Baicheng-You chickens (Figure 1) at the National Breeding Farm in Baicheng County, Aksu, Xinjiang, China. All chickens were feed individually in cages and provided a complete diet during the laying period, with free access to food and water. They followed a standard vaccination schedule. A total of 742 individuals with complete egg-laying records were selected for this study. The first egg laid by each hen was collected and weighed as the first egg weight (FEW). The body weight of the hen at the time when the first egg was laid was recorded as the body weight at first egg (BWFE). The number of eggs laid (EN) was recorded daily from 21 to 52 weeks of age. Based on the egg production rate bar chart (Supplementary Figure S1), the peak egg-laying period was defined as 24–35 weeks of age. The number of eggs laid at 24 weeks was designated as EN1, and the number laid during the peak period was designated as EN2. Additionally, the maximum number of consecutive days of egg laying (MCD) for each hen was calculated based on the egg-laying records.

### 2.3. Sample Collection and Genomic DNA Extraction

Whole blood samples were collected from all experimental chickens at 18 weeks of age, and DNA was extracted using a kit from Tiangen Biochemical Technology Co., Ltd., Beijing, China. DNA degradation was assessed via 0.8% agarose gel electrophoresis, and DNA concentration was precisely quantified using Qubit 3.0 Fluorometer, Thermo Fisher Scientific, Waltham, MA, USA. DNA samples with a total yield exceeding 1.5 μg were selected for library construction.

### 2.4. Reference Genome Mapping and Quality Control

The whole-genome re-sequencing data was mapped to the bGalGal1.mat.broiler.GRCg7b reference genome using BWA-MEM. Subsequent processing, including sorting, duplicate marking, and variant calling, was performed using the Sentieon pipeline (util sort, Dedup, and Haplotyper, respectively). Variant detection was performed on BAM files using Sentieon Haplotyper, producing single-sample variant files in GVCF mode. The GVCFtyper module then conducted joint variant detection on GVCF files from multiple samples, generating multi-sample VCF files. To ensure accuracy in subsequent genetic analyses, quality control (QC) of the SNP data was performed using PLINK (Plink v1.90). Sites with a genotype missing rate exceeding 5% (--geno 0.05) and an allele frequency below 1% (--maf 0.01) were excluded, and samples with a missing rate exceeding 20% (--mind 0.2) were filtered out.

### 2.5. Linkage Disequilibrium

To identify independent variant loci, we used PLINK (v1.90) with the parameter --indep-pairwise 50 5 0.2 for linkage disequilibrium pruning, reducing redundancy and enhancing statistical power. Since the samples were a subset of the genotype data, and GWAS requires identical sample numbers and order between phenotype and genotype files, screening and sorting were necessary. To maintain the integrity and accuracy of the genotype data and prevent SNP quality degradation due to sample size variations, we re-performed quality control using PLINK (v1.90) with --geno 0.05 and --maf 0.01.

### 2.6. Principal Component Analysis

Principal component analysis (PCA) was conducted using PLINK (v1.90) on all autosomal SNPs. The top 20 principal components (PCs) were calculated for each individual, with PC1, PC2, and PC3 plotted on the x-axis and y-axis, respectively. PC significance testing was performed using EIGENSTRAT software (version 6.1.4). The first 10 principal components were extracted as fixed-effect covariates to correct for population structure effects.

### 2.7. Phenotypic Correction and Genetic Parameter Estimation

Prior to genetic analysis, phenotypic data underwent rigorous quality control. Values falling outside the range of mean ±3 standard deviations were excluded. The normality of trait distributions was assessed using the Shapiro–Wilk test. For phenotypes that significantly deviated from normality, rank-based inverse normal transformation was applied to meet the assumptions of parametric analysis.

Genetic parameters for egg production traits were estimated using a linear mixed model implemented in the ASReml-R software package (v4.1.0.176). This model was fitted within the Genomic Best Linear Unbiased Prediction (GBLUP) framework, which utilizes genome-wide markers to construct the relationship matrix, thereby capturing the cumulative effect of all SNPs for accurate genetic parameter estimation. The model was specified as follows:

y = Xb + Z_1_a + Z_2_c + e

In this model:

y is the vector of observed phenotypic values.

X is the design matrix for fixed effects. In our final model, the sole fixed effect was cage row, which was included to statistically control for and correct environmental variation (e.g., gradients in light intensity and temperature) arising from different cage positions.

b is the vector of solutions for the fixed effect (cage row).

Z_1_ and Z_2_ are design matrices for the random effects.

a is the vector of additive genetic effects, distributed as a ~ N(0, Gσ^2^a), where G is the genomic relationship matrix (GRM) derived from the genome-wide SNP data, and σ^2^a is the additive genetic variance.

c is the vector of permanent environmental effects, distributed as c ~ N(0, Iσ^2^c), where I is an identity matrix and σ^2^c is the variance component for the permanent environmental effect.

e is the vector of residual effects, assumed e ~ N(0, Iσ^2^_e).

The genomic heritability (h^2^snps), representing the proportion of phenotypic variance explained by the cumulative effect of all genome-wide SNPs, was calculated as h^2^snps = σ^2^a/(σ^2^a + σ^2^c + σ^2^e).

### 2.8. Genome-Wide Association Analysis

For egg production related traits, we used GEMMA software (version 0.96, https://github.com/genetics-statistics/GEMMA/releases (accessed on 17 June 2025)) to construct a mixed linear model (MLM) for genome-wide association analysis. The model incorporated fixed effects to account for non-genetic variations, including the cage position (as a proxy for light and temperature gradients) and the first ten principal components (PCs) to correct for potential population stratification. The model is as follows:

y = Xα + Zβ + Wμ + ε

In this model, y represents the phenotypic trait; X is the design matrix for fixed effects (cage position and the first 10 PCs); α is the vector of estimated fixed effect parameters; Z is the design matrix for single-nucleotide polymorphisms (SNPs); β represents the SNP effect sizes; W is the design matrix for random effects; μ is the vector of predicted random individual effects; and ε denotes the random residuals.

To control for multiple testing, genome-wide significance thresholds were set using the Bonferroni correction. The number of independent tests (N) was determined from the linkage disequilibrium-pruned SNP set (PLINK parameters: --indep-pairwise 50 5 0.2). The genome-wide and suggestive significance thresholds were set at *p* < 4.45 × 10^−8^ (0.05/N) and *p* < 8.91 × 10^−7^ (1/N), respectively.

### 2.9. Gene Functional Annotation and Enrichment Analysis

We extracted SNP genotypes that showed significant genome-wide associations with various traits and utilized nonparametric tests (Mann–Whitney U test and the Kruskal–Wallis test) to determine the presence of significant phenotypic variation between different genotypes. All analyses were based on the Gallus gallus GRCg7b reference genome assembly. The Variant Effect Predictor (VEP, Ensembl version 91, European Molecular Biology Laboratory’s European Bioinformatics Institute (EMBL-EBI), Hinxton, United Kingdom)) was used to functionally annotate genes within the ±100 kb region of candidate SNPs. Functional enrichment of candidate genes was analyzed using the g:Profiler g:GOSt tool (version e112_eg57_p18_4c72d82, University of Tartu, Tartu, Estonia), with significant terms identified using the g:SCS multiple-testing correction threshold. To annotate potential regulatory elements in non-coding regions, we utilized published human enhancer annotations from the GTEx project [14], mapped their coordinates (hg38) to the chicken genome (GRCg7b) using UCSC LiftOver (version 2024-09-10, UCSC Genome Browser, University of California, Santa Cruz, CA, USA), and focused on those active in reproductive and endocrine-related tissues.

### 2.10. Statistical Fine-Mapping Analysis

To identify putative causal variants within genome-wide significant loci, we performed statistical fine-mapping using the Sum of Single Effects (SuSiE) model, a Bayesian variable selection method implemented in the R package susieR (version 0.14.2) [15]. This approach accounts for linkage disequilibrium (LD) structure and computes the posterior inclusion probability (PIP) for each variant, reflecting its likelihood of being causal. We applied a uniform prior, assuming an equal initial causal probability for all variants within a locus. Results are reported as 95% credible sets, defined as the smallest set of variants that collectively contain at least one true causal variant with a posterior probability ≥ 95%. To explore potential links between GWAS signals and gene regulation, we further integrated fine-mapping results with regulatory quantitative trait loci (QTLs) from the chicken GTEx project. Using a Bayesian colocalization framework [16], we evaluated statistical evidence for shared causal variants between traits. A posterior probability of colocalization (PP4) greater than 0.80 was set as the threshold for strong evidence of colocalization. Variants located within fine-mapping credible sets and showing colocalization with regulatory QTLs were prioritized as high-confidence candidate causal variants for subsequent analysis.

## 3. Results

### 3.1. Phenotypic Statistics of Egg-Laying Related Traits in Baicheng-You Chicken

Descriptive statistical analysis of the egg production traits of Baicheng-You chickens is shown in Table 1. As shown in Table 1, We collected egg-laying-related traits from 742 Baicheng-You hens, including first egg weight (FEW), body weight at first egg (BWFE), total number of eggs at 24 weeks (EN1), total number of eggs from 24 to 35 weeks (EN2), and maximum consecutive days of egg laying (MCD). After filtering outliers, an average of 731 valid records per trait were retained. The average FEW was 39.58 g, with a range from 26.64 g to 52.14 g. The average BWFE was 1866.32 g, and the average MCD was 11.72 days. The mean numbers of eggs for EN1 and EN2 were 5.50 and 63.97, respectively, the bar charts, scatter plots, and Q-Q plots for each trait are shown in Supplementary Figure S2.

We evaluated the egg-laying performance at different stages: at 24 weeks and from 24 to 35 weeks, representing the peak laying period. Notably, the phenotypic variation coefficients for MCD and EN1 were 57.24% and 22.62%, respectively. These findings suggest significant potential for improving the initial laying and continuous egg-laying performance of Baicheng-You Chickens, providing a foundation for enhancing their production performance.

### 3.2. Variation Detecting and Estimation of Genetic Parameters for Egg-Laying Traits

**Figure 2:**
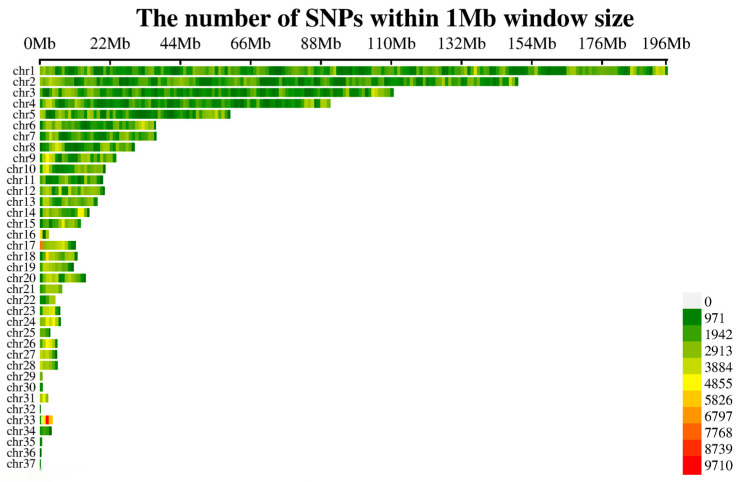
SNP density map.

We obtained whole-genome resequencing data from 742 Baicheng-You chickens to accurately detect genome-wide variations. The total sequencing data amounted to 2.37 Tb, with an average of 37.70 million paired-end reads per individual. The sequencing data was aligned to the chicken reference genome (GRCg7b) with an average alignment rate of 99.73%. The average sequencing depth was >5×. The average base quality score (Q30) across all samples was 93.26%. Using the GATK standard pipeline, we detected 28,597,909 raw variants, comprising 5,231,654 indels and 23,366,255 SNPs. After stringent quality control, approximately 92.8% of the raw variants were filtered out, resulting in 2,053,663 high-quality SNPs (SNP density plot is shown in Figure 2) that were retained for subsequent genetic parameter estimation and genome-wide association analysis. The quality of this final SNP set was rigorously validated, demonstrating a high mean genotyping rate (call rate) of 0.97 and a transition/transversion (Ti/Tv) ratio of 2.596, which is within the expected range for high-quality data in chickens.

As presented in Table 2, Genetic parameter estimation for egg-laying traits in Baicheng-You chickens revealed heritabilities of 0.494 for FEW, 0.592 for BWFE, 0.489 for MCD, 0.568 for EN1, and 0.382 for EN2, indicating medium to high heritability. These findings suggest that genetic improvement is valuable for enhancing egg-laying performance. Trait correlations revealed complex relationships; for instance, MCD was positively correlated with both EN1 and EN2, with coefficients exceeding 0.585, indicating a strong association between maximum consecutive days of egg production and egg number. The positive correlation between BWFE and FEW (r = 0.240) suggests that initial egg weight may influence subsequent egg weight. Conversely, EN1 and EN2 was negatively correlated with BWFE, potentially reflecting a balance between early egg production and the physiological state of the hen. Further investigation into the underlying biological mechanisms is warranted. These genetic parameters provide crucial insights into the genetic basis of egg production traits in Baicheng-You chickens, laying a theoretical foundation for enhancing egg production performance through targeted genetic selection.

### 3.3. Genome-Wide Association Analysis of Egg Production Traits

We initially performed principal component analysis (PCA) on 742 Baicheng-You chickens. The first three principal components were visualized and confirmed the absence of significant population stratification (Supplementary Figure S3), indicating the suitability of this population for genome-wide association studies. To rigorously control for potential confounding due to population structure and minimize false positives, the first ten principal components were incorporated into the mixed linear model as fixed-effect covariates.

**Figure 3:**
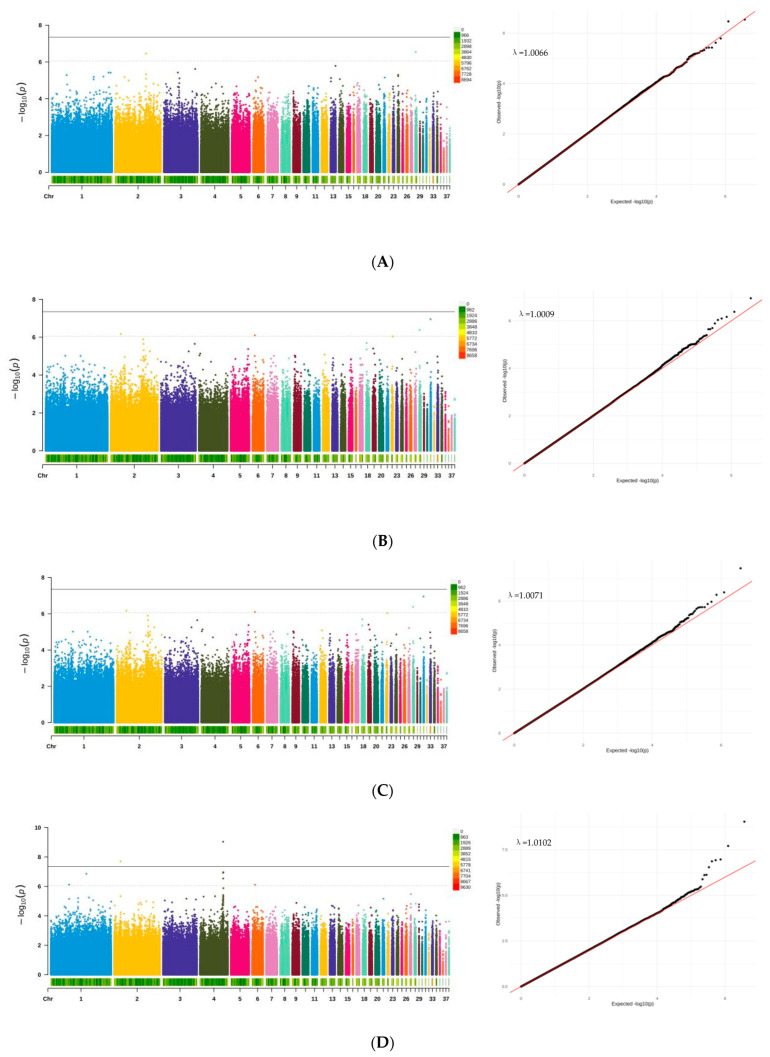

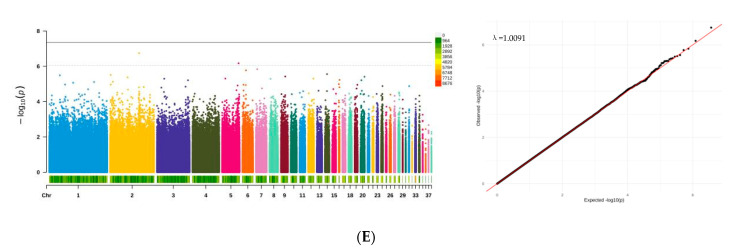
Genome-wide association study results for egg-laying traits in Baicheng-You chickens. Manhattan plots (left) and quantile-quantile (QQ) plots (right) are shown for: (**A**) number of eggs at 24 weeks (EN1), (**B**) number of eggs from 24 to 35 weeks (EN2), (**C**) first egg weight (FEW), (**D**) body weight at first egg (BWFE), and (**E**) maximum consecutive laying days (MCD). In the Manhattan plots, the red horizontal line indicates the genome-wide significance threshold (*p* < 4.45 × 10^−8^), and the blue dashed line indicates the suggestive significance threshold (*p* < 8.91 × 10^−7^). The reference genome assembly used was GRCg7b.

We then conducted univariate genome-wide association studies (GWAS) for egg production using mixed linear models. To further assess the robustness of the GWAS model and the control of spurious associations, we calculated the genomic control inflation factor (λ). The λ values for all traits ranged from 1.0009 to 1.0102 (Supplementary Table S1), indicating minimal genomic inflation and effective control for population stratification and other potential confounders. According to Table 3, 19 genome-wide single-nucleotide polymorphisms (SNPs) were identified, including 3 genome-wide significant SNPs (*p* < 4.45 × 10^−8^) and 16 genome-wide suggestive SNPs (*p* < 8.91 × 10^−7^), distributed across 9 chromosomes (GGA1, GGA2, GGA3, GGA4, GGA5, GGA6, GGA8, GGA28, and GGA31). Notably, rs28:1645873 showed suggestive or significant associations with both EN1 and EN2. The Manhattan plots and Q-Q plots for each trait are displayed in Figure 3A–E. Table 3 lists all SNP information. In the EN1 stage, 2 genome-wide suggestive SNPs were located on chromosomes GGA2 and GGA28. In the EN2 stage, 4 genome-wide suggestive SNPs were identified on chromosomes GGA2, GGA6, GGA28, and GGA31. For the FEW trait, 3 genome-wide SNPs (one significant and two suggestive) were found on chromosomes GGA3, GGA4, and GGA8. 8 genome-wide SNPs (two significant and six suggestive) were identified for the BWFE trait on chromosomes GGA1, GGA2, GGA4, and GGA6. Two genome-wide suggestive SNPs were detected for the MCD trait on chromosomes GGA2 and GGA5. The contribution rate of all SNPs for the phenotypic variation explained (PVE) ranged from 2.28% to 4.12%, despite the modest contribution of individual SNPs, the high genomic heritability (0.382–0.592) estimated via the GBLUP model underscores the substantial cumulative predictive ability of all SNPs for these traits. We used VEP to annotate the genes potentially regulated by the significantly associated SNPs. Among them, 9 were located within genes and 10 were located in intergenic regions. To further investigate the regulatory effects of intergenic variants on traits and genes, we annotated regulatory elements using chicken GTEx data. Two significantly associated SNPs were annotated as enhancer regions in the chicken genome. Specifically, 2:31393713 is located 12.7 kb upstream of the *SKAP2* gene, and 5:56013517 is located 17.1 kb upstream of the *SAMD4A* gene. Although queries in existing avian eQTL databases did not yield significant evidence, the enhancer annotations of these loci and their strong associations with GWAS suggest their potential regulatory roles in reproductive traits. The specific data are shown in Supplementary Table S2. The quantile-quantile plots for all traits demonstrated a clear alignment along the diagonal line, with a slight upward tilt at the tail, indicating effective control of false positives and the reasonableness of the model.

### 3.4. Function Enrichment Analyses of Associated Genes

**Figure 4:**
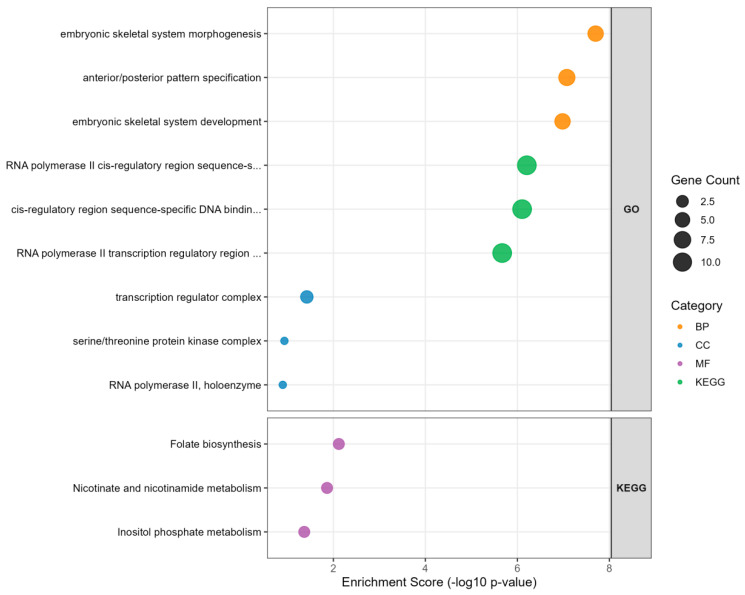
Most significantly enriched pathways.

In this study, a genome-wide analysis identified 120 candidate genes significantly associated with egg production traits in Baicheng-You chickens. To elucidate the potential biological functions of these genes, we conducted Gene Ontology (GO) and Kyoto Encyclopedia of Genes and Genomes (KEGG) pathway enrichment analyses. GO and KEGG enrichment analysis results showed (Figure 4), GO enrichment analysis revealed significant enrichment in biological processes such as embryonic skeletal system morphogenesis, anterior/posterior pattern specification, RNA polymerase II cis-regulatory region sequence-specific DNA binding, and transcription regulator complex. KEGG pathway analysis indicated that the candidate genes were most significantly enriched in folate biosynthesis. Additionally, pathways such as nicotinate and nicotinamide metabolism, and inositol phosphate metabolism were significantly enriched. These pathways may regulate egg production by influencing processes such as follicle development and steroid hormone synthesis. Complete results are shown in Supplementary Table S3.

### 3.5. Fine-Mapping for Traits

**Figure 5:**
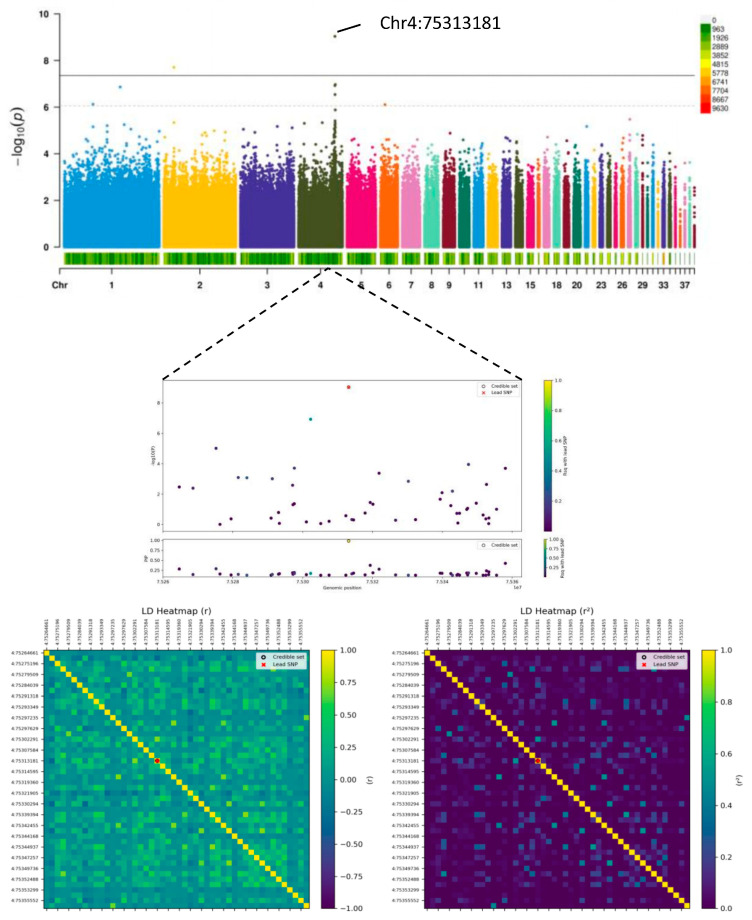
Statistical fine-mapping and linkage disequilibrium (LD) analysis of the significant locus on chromosome GGA4 associated with body weight at first egg (BWFE). The upper panel shows the posterior inclusion probability (PIP) for each variant in the region, with the lead SNP (4:75313181) highlighted. The lower panel displays the pairwise LD (r^2^) between the lead SNP (indicated by the red arrow) and surrounding variants. Analysis was based on the GRCg7b genome assembly.

Focusing on the candidate QTL regions listed in Table 3, fine-mapping analysis calculated the posterior causal probability (PPC) for each variant and gene, identifying candidate genes consistent with the GWAS results. 24 genes located within 50 kb of the GWAS signals were successfully fine-mapped, including *STEAP4*, *LCORL*. As shown in Figure 5, the variant at chr4:75313181 exhibited a significant association with BWFE, and was in strong linkage disequilibrium with surrounding SNPs. These results demonstrate that the variants identified through genome-wide association analysis are accurate and provide valuable data for developing molecular markers for reproductive traits (The complete results are presented in Supplemental Figure S4).

## 4. Discussion

The Baicheng-You chicken is a highly specialized germplasm resource in Xinjiang, China, which has not yet undergone systematic genetic evaluation or analytical marker discovery. Thie study is the first to focus on the egg production performance of Baicheng-You chicken using population genome resequencing data to estimate genetic parameters and identify potential molecular markers related to egg production traits. The genetic data and candidate variants we provide serve as valuable resources for the further development and utilization of this breed.

Decades of research have proven that estimating genetic parameters using genome-wide SNP data is accurate, and genome-wide association analysis is effective for identifying loci significantly associated with traits for molecular breeding [17]. Egg production is a crucial economic trait in modern poultry breeding, directly impacting the economic benefits and market competitiveness [18]. It encompasses multiple key parameters, including egg weight at the start of lay, body weight at the start of lay, egg production, and egg production persistence, forming a vital foundation for evaluating poultry performance [19]. We used whole-genome sequencing technology to analyze egg production traits in the Baicheng-You chicken. Compared to traditional genotyping methods, this approach provides higher accuracy and coverage, enabling comprehensive identification of single nucleotide polymorphisms (SNPs) across the genome [20,21]. We generated high-depth whole-genome resequencing data from 742 Baicheng-You chickens, identifying 2,053,663 high-quality SNPs for five key egg production traits, including egg weight at the start of lay, body weight at the start of lay, and the number of eggs laid during peak production. This represents the first large-scale presentation of genetic resources from this unique breed. Our study is the first systematic assessment of genetic parameters for egg production traits in the Baicheng-You Chicken. The results showed heritabilities ranging from 0.382 to 0.592, indicating medium to high levels, which suggests significant potential for genetic improvement. Notably, traits such as MCD, EN1, and EN2 exhibited high genetic and phenotypic correlations (0.384–0.876), consistent with previous studies, suggesting a shared genetic regulatory network [22,23]. Interestingly, while FEW and BWFE traits exhibited high heritabilities, their correlations with other traits were relatively low, we hypothesize this is due to distinct endocrine controls, where the initiation of FEW and BWFE is governed by puberty-specific pathways, separate from the homeostatic mechanisms sustaining long-term production [24]. This genetic decoupling is advantageous for breeding, and applying a multi-trait GWAS may reveal latent pleiotropy to further optimize selection for both trait groups [25].

Through genome-wide association studies (GWAS), we identified 19 single nucleotide polymorphisms (SNPs) with suggestive (*p* < 8.91 × 10^−7^) or significant (*p* < 4.45 × 10^−8^) associations across the genome. We annotated 36 genes within a 200 kb region, including *LCORL*, *ADAM22*, *ENSGALG00010011716*, *NCAPG*, and *FAM184B*, potentially associated with these SNPs. *FAM184B* has been repeatedly associated with important economic traits in livestock, such as litter size in sheep [26], carcass weight in Korean beef [27], and organ weight in Simmental cattle [28]. In poultry, Yang et al. [29] identified FAM184B as associated with body weight and egg quality in Wenshui Green Shell Chickens. Lyu et al. [30] highlighted its potential role in growth and muscle mass in chickens. Zhang et al. [31] and FAN et al. [32] found *FAM184B* linked to reproductive traits in Jinghai Yellow Chickens. Luo et al. [33] identified it as affecting body weight in Wenchang Chickens. Collectively, these findings suggest that *FAM184B* may be an important regulator of growth, development, reproductive performance, and carcass traits in various livestock and poultry species. *NCAPG* is part of the condensin complex, involved in chromatin structure maintenance and chromosome segregation during cell division [34]. Ma et al. [35] found *NCAPG* might affect egg weight. Yi et al. [36] suggested it influences longitudinal egg weight through amino acid substitution. Shen et al. [37] linked *NCAPG* to oviduct development, affecting cell survival, appetite, and growth control. Sun et al. [38] noted its impact on eggshell quality. Barkova et al. [39] associated *NCAPG* with egg weight. In mammals, it is linked to body size traits [40], fetal growth [41], and myogenesis in cattle [42]. In summary, both *FAM184B* and *NCAPG* are located within significant QTLs and represent high-priority candidate genes for egg production traits in our population. While our data and the literature establish a strong association, future functional studies are needed to confirm their causal roles. Based on their proposed functions, we hypothesize that these genes influence egg production by regulating the development and function of reproductive tissues, such as the ovary and oviduct. A direct test of this hypothesis would be to investigate their spatiotemporal expression patterns in the hen reproductive tract throughout the laying cycle.

To further elucidate the functions of these candidate genes, we conducted GO and KEGG enrichment analyses. The results revealed a significant over-representation of terms related to embryonic skeletal system morphogenesis (GO:0048704), transcription regulator complex (GO:0005667), RNA polymerase II cis-regulatory region sequence-specific DNA binding (GO:0000978), and folate biosynthesis (gga00790). The enrichment of embryonic skeletal system morphogenesis (GO:0048704) is critically relevant to egg-laying physiology. In laying hens, the medullary bone, a component of the skeletal system, undergoes continuous remodeling to serve as a labile calcium reservoir. We hypothesize that the up-regulation of genes involved in this process (e.g., members of the *HOXA* family) facilitates enhanced calcium mobilization for eggshell calcification, thereby directly influencing eggshell quality and strength [43]. Furthermore, the significant enrichment of the folate biosynthesis pathway (gga00790) suggests a key role in supporting high rates of oogenesis. Folate is indispensable for nucleic acid synthesis and methylation reactions [44]. An elevated activity in this pathway is presumed to underpin the rapid cell proliferation required for follicular development in the ovary, potentially leading to an increased laying rate. Finally, the strong signal in transcriptional regulatory functions (GO:0005667, GO:0000978) underscores the importance of precise gene expression control [45]. These terms encompass transcription factors that likely orchestrate the expression of downstream genes governing steroid hormone synthesis, oviductal function, and overall reproductive system maintenance. Enrichment analysis results indicated widespread involvement of *HOXA* family genes, which are central to many of these processes. Research by Gligorov et al. [46] demonstrated that regulatory mutations in the *HOX* homeotic gene Abdominal-B (*Abd-B*) disrupt the function of secondary cells in the Drosophila accessory gland. In vertebrates, the orthologs of *Abd-B*, namely *HOXA9-13*, regulate hindlimb and genital development. Liao et al. [47] selected five early developmental stages of White Leghorn chickens for transcriptome analysis and identified *Zf-C2H2*, *HOX*, and *bHLH* as three dominant transcription factor families emerging during early embryogenesis. Villaescusa et al. [48] found that *HOX* and *HOX* cofactor genes are involved in oocyte maturation and development, as well as granulosa cell differentiation. Additionally, polymorphisms in the *Hoxa2b* gene were significantly associated with embryonic mortality [49]. These studies collectively highlight the crucial roles of *HOXA* family genes in animal embryonic development, reproductive organ formation, and oocyte maturation. In Baicheng-You chickens, *HOXA* family genes may ultimately influence egg-laying performance by precisely regulating key processes such as follicular development, steroid hormone synthesis, and eggshell quality. Collectively, these enriched terms implicate a coordinated network where developmental, metabolic, and regulatory processes converge to determine egg-laying performance.

## 5. Conclusions

This study is the first to estimate genetic parameters and conduct genome-wide association analysis for egg-laying traits in the Baicheng-You chicken, a specialty breed in Xinjiang. We identified 19 SNPs and 120 protein-coding genes significantly associated with traits such as weight at the start of laying, egg weight at the start of laying, maximum consecutive days of laying, number of eggs laid at 24 weeks, and peak laying period. *NCAPG*, *FAM184B*, and *HOXA* family are closely linked to growth, follicular development, embryonic development, and oviduct development. These findings provide new insights into developing molecular markers for reproductive performance in Baicheng-You chicken. It should be noted that these findings, derived from a single population, require further validation in independent cohorts to confirm their generalizability. Future efforts should focus on such replication and on functional studies to elucidate the underlying biological mechanisms. This study offers valuable genetic resources for understanding the genetic basis of egg-laying traits and provides scientific and technological support for the conservation, development, and utilization of this breed.

## Figures and Tables

**Table 1:** Descriptive statistics of egg production characteristics of Baicheng-You hens.

Traits	Count	Mean	Std	CV	Min	Max
FEW	733	39.58	3.96	10.01	26.64	52.14
BWFE	734	1866.32	219.83	11.78	1275.89	2521.20
MCD	729	11.72	6.71	57.24	3	39
EN1	721	5.50	1.24	22.62	1	8
EN2	737	63.97	7.73	12.09	40	85

Count is the sample size, Mean is the average value, Std is the standard deviation, The coefficient of variation (CV) is defined as the ratio of the standard deviation to the mean, Min is the minimum value, Max is the maximum value.

**Table 2:** Genetic parameters estimation of egg production traits in Bicheng-You chicken.

Traits	FEW	BWFE	MCD	EN1	EN2
FEW	0.494	0.240	−0.001	−0.034	0.002
BWFE	0.361	0.592	0.042	−0.097	−0.084
MCD	−0.013	0.070	0.489	0.384	0.669
EN1	−0.066	−0.161	0.585	0.568	0.480
EN2	−0.012	−0.162	0.876	0.861	0.382

The diagonal is heritability, the lower triangle is genetic correlation, and the upper triangle is phenotypic correlation.

**Table 3:** SNPs of egg laying characteristics in Bicheng-You chickens.

Traits	RS	ALT/REF	AF	Beta ± SE	*p*_Wald	PVE (%)
EN1	2:101153947	A/T	0.025	0.68 ± 0.13	3.45 × 10^−7^	2.28
	28:1645873	A/C	0.082	0.39 ± 0.08	2.91 × 10^−7^	2.34
EN2	2:32393713	T/G	0.110	−0.40 ± 0.08	6.81 × 10^−7^	3.13
	6:7641679	G/C	0.031	−0.68 ± 0.14	7.97 × 10^−7^	2.78
	28:1645873	A/C	0.082	0.42 ± 0.08	4.17 × 10^−7^	2.62
	31:521682	C/T	0.020	−0.88 ± 0.17	1.12 × 10^−7^	3.07
FEW	3:51719751	A/G	0.016	0.90 ± 0.18	5.26 × 10^−7^	2.56
	8:15494087	T/C	0.054	−0.52 ± 0.10	4.07 × 10^−7^	2.78
	4:71886853	C/G	0.025	0.72 ± 0.13	3.29 × 10^−8^	2.54
BWFE	1:59463692	C/T	0.025	0.74 ± 0.15	7.49 × 10^−7^	2.67
	1:115602864	C/T	0.042	0.62 ± 0.12	1.36 × 10^−7^	3.10
	2:20994158	A/G	0.015	1.13 ± 0.20	1.96 × 10^−8^	3.79
	4:75313181	T/A	0.356	0.30 ± 0.05	9.18 × 10^−10^	4.12
	4:75302291	T/C	0.387	0.26 ± 0.05	1.18 × 10^−7^	3.21
	4:75541018	G/A	0.394	0.25 ± 0.05	2.88 × 10^−7^	3.06
	4:76087313	A/G	0.466	−0.25 ± 0.05	1.08 × 10^−7^	3.03
	6:9691566	T/A	0.343	0.25 ± 0.05	7.76 × 10^−7^	2.83
MCD	2:98466537	T/C	0.252	−0.29 ± 0.06	1.79 × 10^−7^	3.27
	5:56013517	C/T	0.018	0.84 ± 0.17	6.71 × 10^−7^	2.51

This table presents single nucleotide polymorphisms (SNPs) that reached genome-wide significant or suggestive significant levels in the genome-wide association study. RS: SNP identifier; ALT/REF: Effect allele/Reference allele; AF: Effect allele frequency; Beta ± SE: Effect size and its standard error; *p*_wald: *p*-value from the Wald test; PVE (%): Percentage of phenotypic variance explained by the SNP. The genome-wide significance threshold was set at *p* < 4.45 × 10^−8^, and the suggestive significance threshold was set at *p* < 8.91 × 10^−7^.

## Data Availability

All data generated or analyzed during this study are included in this published article and its Supplementary information files.

## Supplementary Materials

The following supporting information can be downloaded at: https://www.mdpi.com/article/10.3390/ani15233360/s1, Supplementary Figure S1: Baicheng-You Chickens Egg Production Rate Curve Chart; Supplementary Figure S2: Scatter plot, bar chart, and Q-Q plot of egg-laying traits in Baicheng-You chickens; Supplementary Figure S3: Principal component analysis; Supplementary Figure S4: A: Fine mapping and LD analysis of significant SNP loci on GGA4 for FEW trait. B: Fine mapping and LD analysis of significant SNP loci on GGA2 for BWFE trait. Supplementary Table S1: The genomic inflation factor (λ) for each phenotype is as follows; Supplementary Table S2: Candidate Gene Information for Egg-Laying Traits in Baicheng-You Chickens; Supplementary Table S3: Candidate gene enrichment analysis.
