# Supplementary material for: Genetic Basis of Egg Production in Baicheng-You Chickens: A Genome-Wide Association Study
Source: Animals (Basel). 2025 Nov 21;15(23):3360. doi: 10.3390/ani15233360 (PMC12691223; doi:10.3390/ani15233360)
Supplement: Supplementary file 1 [file animals-15-03360-s001.zip › Supplementary Figure.pdf]

Supplementary Figure S1 Baicheng-You Chickens Egg Production Rate Curve Chart

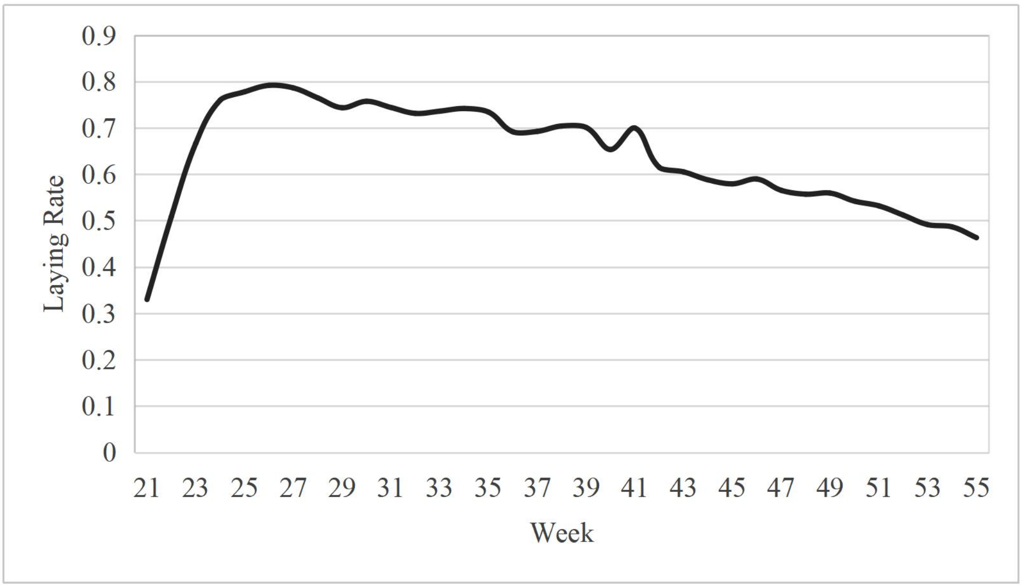


Supplementary Figure S1. Egg production rate curve of Baicheng-You chickens from 21 to 52 weeks of age. The x-axis represents the age of hens in weeks. The y-axis represents the egg production rate (%).

Supplementary Figure S2 Scatter plot, bar chart, and Q-Q plot of egg-laying traits in Baicheng-You chickens

A

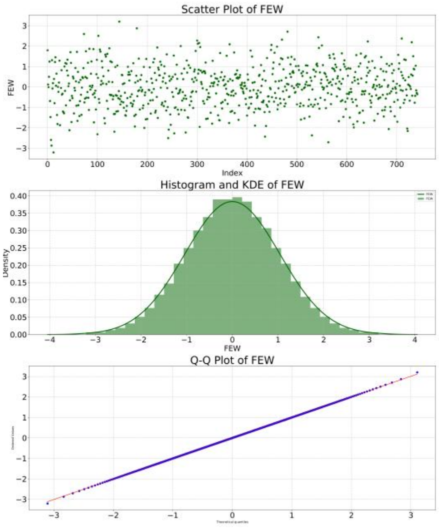


B

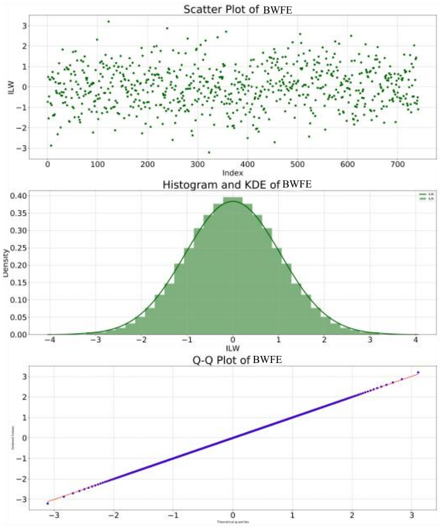


C

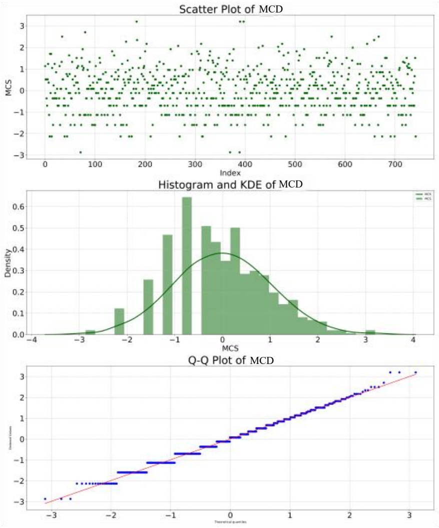


D

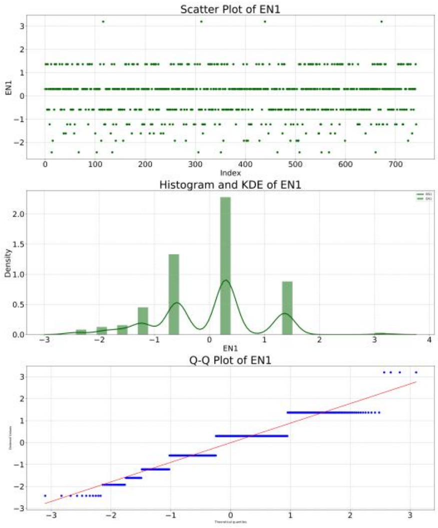


E

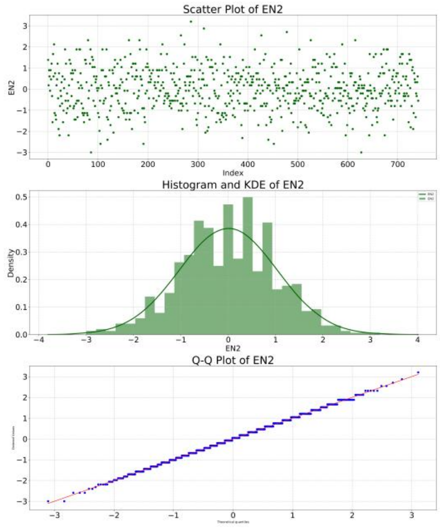

Supplementary Figure S2. Distribution and normality assessment of the five egg-laying traits. For each trait (A, First Egg Weight, FEW; B, Body Weight at First Egg, BWFE; C, Maximum Consecutive Days, MCD; D, Number of Eggs at 24 weeks, EN1; E, Number of Eggs from 24-35 weeks, EN2), three panels are presented from left to right: A histogram with a fitted normal distribution curve (showing the frequency distribution of the raw phenotypic values), a scatter plot of the raw phenotypic values, and a Q-Q (Quantile-Quantile) plot for assessing normality (where points aligning with the dashed line indicate a normal distribution).

Supplementary Figure S3 Principal component analysis

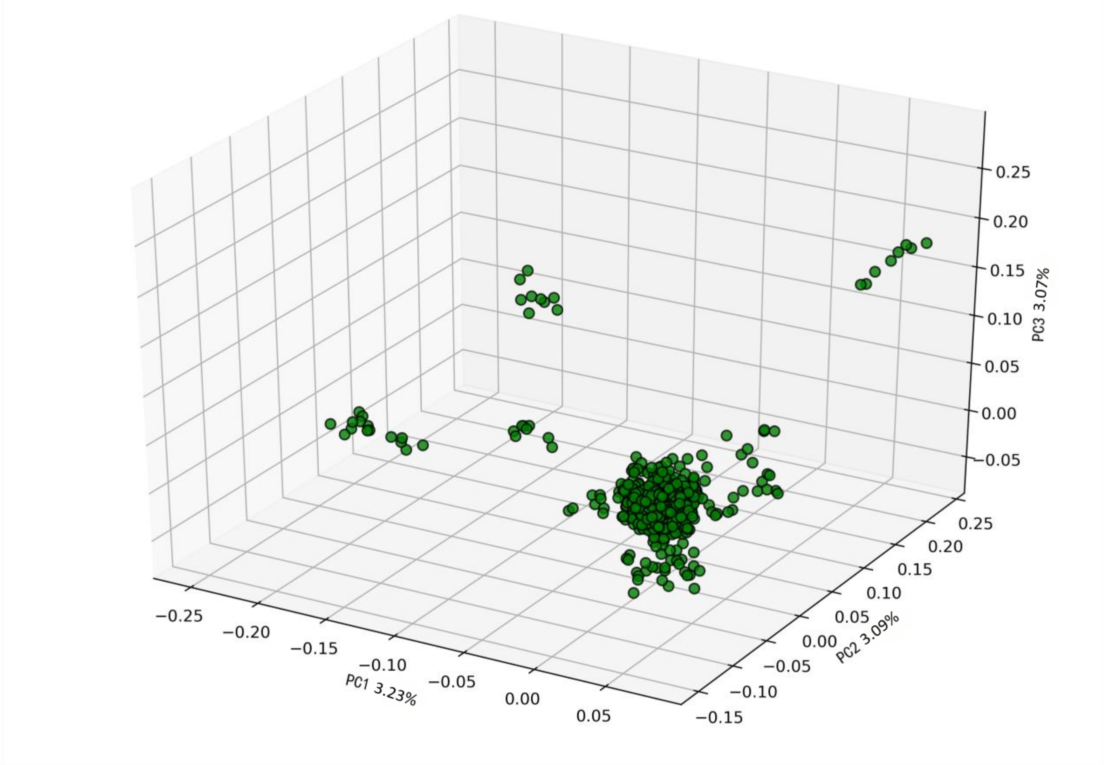


Supplementary Figure S3. Principal component analysis (PCA) of 742 Baicheng-You chickens based on genome-wide autosomal SNPs. The 3D scatter plot shows the distribution of individuals along the first three principal components (PC1, PC2, and PC3). The percentages on the axes indicate the proportion of total genetic variance explained by each component. The analysis confirms the absence of significant population stratification within the flock, validating its suitability for genome-wide association analysis.

Supplementary Figure S4 A:Fine mapping and LD analysis of significant SNP loci on GGA4 for FEW trait. B:Fine mapping and LD analysis of significant SNP loci on GGA2 for BWFE trait.

A

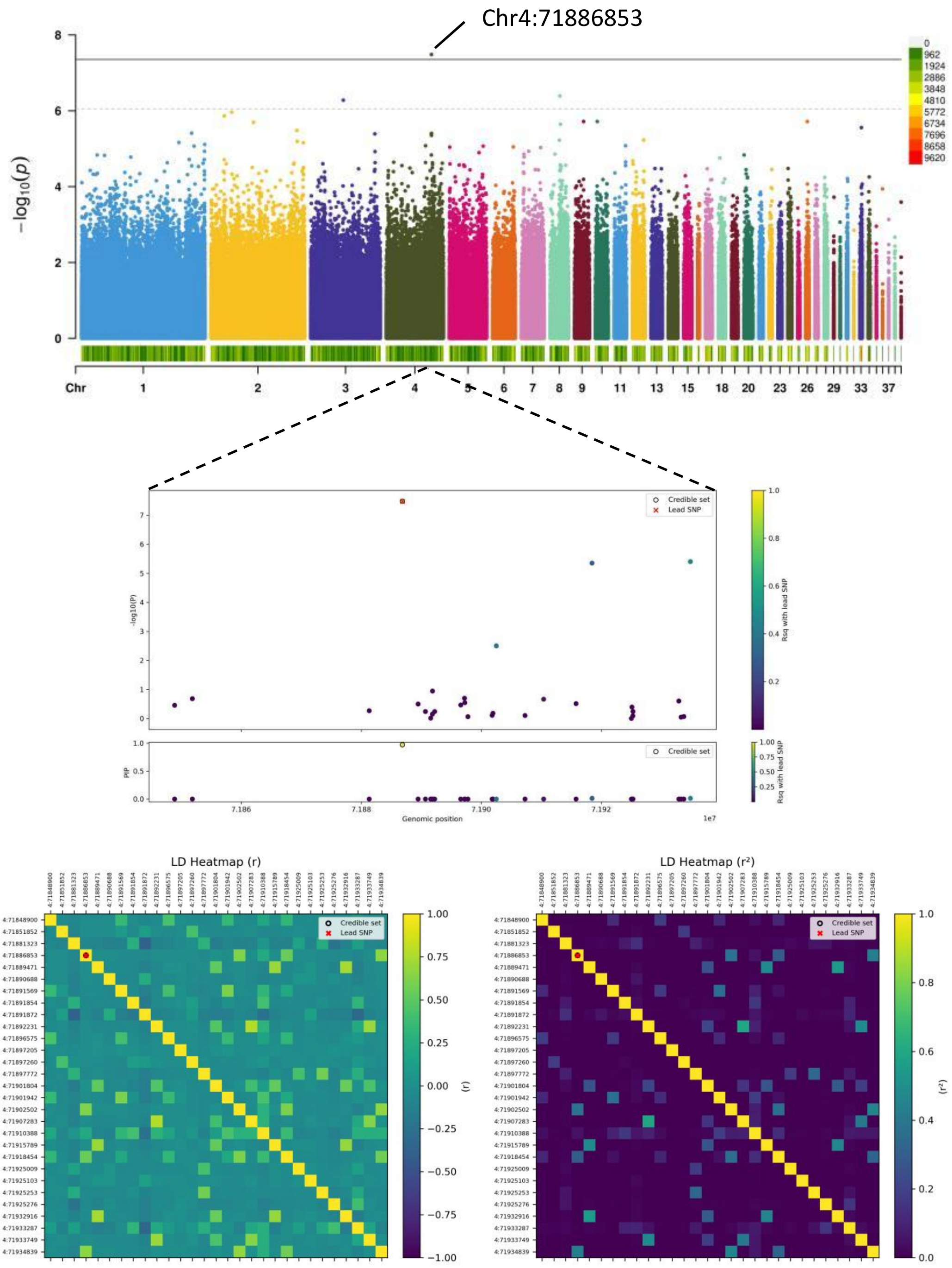

B

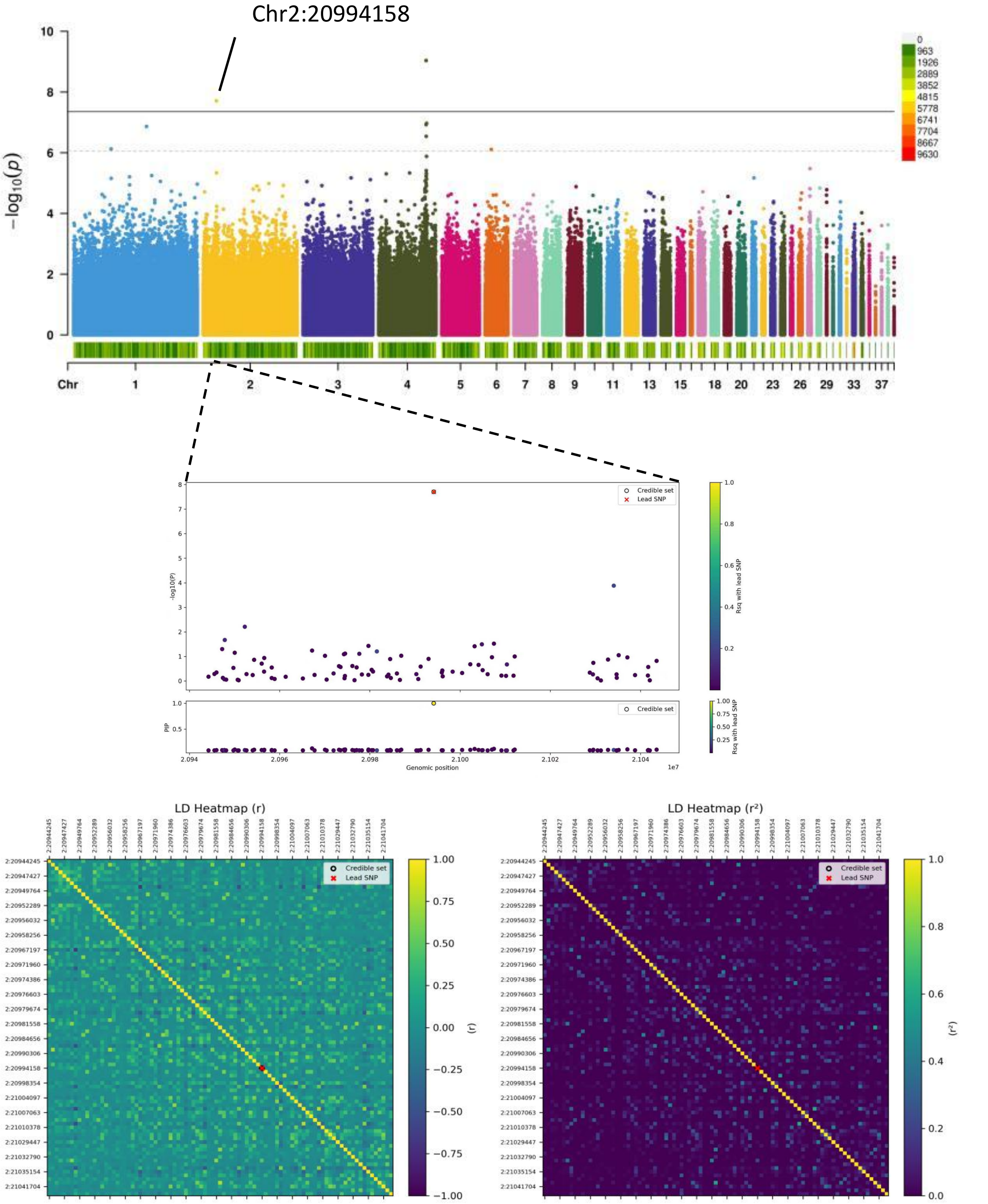


Supplementary Figure S4. Statistical fine-mapping and linkage disequilibrium (LD) analysis of genome-wide significant loci. (A) For the FEW (First Egg Weight) locus on chromosome 4. (B) For the BWFE (Body Weight at First Egg) locus on chromosome 2. For each locus, the top panel is a regional Manhattan plot showing the association signals; the middle panel displays the posterior inclusion probability (PIP) for each variant from the fine-mapping analysis (SuSiE), with the lead SNP and its 95% credible set highlighted; the bottom panel is an LD heatmap (measured by r²) relative to the lead SNP (indicated by a red arrow), illustrating the correlation structure of the region. All genomic coordinates are based on the GRCg7b assembly.
